# The Impact of Multifaceted Osteoporosis Group Education on Patients' Decision-Making regarding Treatment Options and Lifestyle Changes

**DOI:** 10.1155/2018/9703602

**Published:** 2018-03-26

**Authors:** Annesofie L. Jensen, Gitte Wind, Bente Lomholt Langdahl, Kirsten Lomborg

**Affiliations:** ^1^Department of Endocrinology and Internal Medicine, Aarhus University Hospital, Tage-Hansen Gade 2, 8000 Aarhus C, Denmark; ^2^Faculty of Health, Department of Public Health, Aarhus University, Building 1633, Høegh-Guldbergs Gade 6A, 8000 Aarhus C, Denmark; ^3^Department of Culture and Society, Anthropology and Ethnography, Aarhus University, Mosegaard Alle 20, 8270 Højbjerg, Denmark; ^4^Institute of Nursing, Metropolitan University College, Tagensvej 86, 2200 Copenhagen N, Denmark; ^5^Department of Clinical Medicine, The Research Programme in Patient Involvement, Aarhus University, Norrebrogade 44, Building 12A, 8000 Aarhus, Denmark

## Abstract

**Introduction:**

Patients with chronic diseases like osteoporosis constantly have to make decisions related to their disease. Multifaceted osteoporosis group education (GE) may support patients' decision-making. This study investigated multifaceted osteoporosis GE focusing on the impact of GE on patients' decision-making related to treatment options and lifestyle.

**Material and Methods:**

An interpretive description design using ethnographic methods was utilized with 14 women and three men diagnosed with osteoporosis who attended multifaceted GE. Data consisted of participant observation during GE and individual interviews.

**Results:**

Attending GE had an impact on the patients' decision-making in all educational themes. Patients decided on new ways to manage osteoporosis and made decisions regarding bone health and how to implement a lifestyle ensuring bone health. During GE, teachers and patients shared evidence-based knowledge and personal experiences and preferences, respectively, leading to a two-way exchange of information and deliberation about recommendations. Though teachers and patients explored the implications of the decisions and shared their preferences, teachers stressed that the patients ultimately had to make the decision. Teachers therefore refrained from participating in the final step of the decision-making process.

**Conclusion:**

Attending GE has an impact on the patients' decision-making as it can initiate patient reflection and support decision-making.

## 1. Introduction

In today's healthcare system, patients are expected to play an active role and take responsibility for their own health [1, 2]. In light of this development, disease-specific group education (GE) has become an integral and continuing part of healthcare provision [3] and a recommended way to encourage patients to become active participants in their own care [4–6]. Active participation includes making decisions about medical treatment and learning how to make lifestyle changes. However, little is known about participation in multifaceted GE and how it impacts patients' decision-making regarding treatment options and lifestyle changes.

The constant need to make health decisions is evident for patients with the chronic disease osteoporosis [7]. These patients face numerous self-care decisions, for example, whether to take medicine and to start doing weight-bearing exercises. In Denmark, where this study was performed, patients with osteoporosis usually consult their physician or general practitioner to discuss and evaluate the treatment within the first year after starting treatment. Afterwards, treatment is evaluated every 2-3 years; hence, making decisions on how to manage osteoporosis in daily life relies heavily on the patient.

In the encounter between patients and physicians, decision-making is described as an iterative process including three steps: (1) information exchange, (2) deliberation about options, and (3) deciding on treatment to implement [8–10]. The paternalistic, the informed, and the shared decision-making models are commonly used to describe the steps of the decision-making process [8]. In the decision-making process, the question that arises is which kind of support the patients need to make decisions about their treatment and health? Decision support [11] typically involves a combination of consultation, counselling [12], and decision aids, [13] with the overall aim of making the decision that needs to be made explicit, providing information about the options and outcomes, and clarifying personal values.

Research on patients with osteoporosis and decision support has focused on the development and effect of decision aids [14, 15]. Studies have shown that decision aids increase patients' knowledge of options for managing osteoporosis and help them clarify their own preferences [16, 17]. A systematic review found that tools, especially those including reminders and education support, may reduce fracture risk by increased use of osteoporosis medicine leading to increase in bone mineral density (BMD) [18]. A study of patients with osteoporosis fractures and their decisions about taking prescribed osteoporosis medication revealed that regardless of whether the decision was easy or difficult to make, patients stated that the decision was not permanent as a number of circumstances could cause them to change it again [7].

The majority of research on decision-making has focused on the types and effect of decision support in the one-on-one encounter between patients and physicians in the context of acute disease [12, 19, 20]. The context of GE differs from the traditional patient-physician encounter. Typically, it is specified as encounters between two or more patients and several different healthcare professionals who may not be aware of the individual patient's condition and situation before the encounter [21]. In this paper, we investigated multifaceted osteoporosis GE as a possible setting for decision-making, focusing specifically on how attending GE impact on patients' decision-making regarding treatment options and lifestyle changes.

## 2. Participants and Methods

To understand decision-making in the context of GE, we conducted an ethnographic field study [22] using interpretive description as our overall methodology [23–25]. Interpretive description is a qualitative inductive research strategy suitable for complex clinical health studies [24]. The fieldwork was conducted during patients' participation in GE; in addition to participant observation, the fieldwork also included patient interviews.

### 2.1. Setting

**Figure 1:**
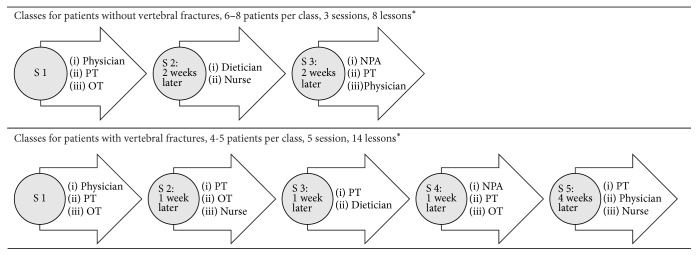
Multidisciplinary osteoporosis group educational programmes: sessions, lessons, and teachers. ^*∗*^Length of lessons is 45 minutes; NPA, National Patients Association; PT, physiotherapist; OT, occupational therapist; S, session.

The fieldwork took place from August 2011 to April 2013 at the endocrinology outpatient clinic of Aarhus University Hospital, Denmark, where structured multifaceted osteoporosis GE has been available since 2003. The clinic offers two different GE programmes: one for patients without vertebral fractures and one for patients with vertebral fractures.  Figure 1 outlines the organisation of two GE programmes and the contribution of the different teachers. The GE programme for patients without fractures comprised 3 sessions of 2-3 lessons each. Physicians and physiotherapists contributed 2 lessons each and occupational therapist, dietician, nurse, and a representative from the national patient organisation contributed 1 lesson each. The GE programme for patients with fractures comprised 5 sessions of 2-3 lessons each. Physiotherapists contributed 5 lessons, occupational therapists contributed 3 lessons, physicians and nurses contributed 2 lessons each and dietician, and a representative from the national patient organisation contributed 1 lesson each. Approximately 90 patients divided into 18 classes (eight classes for patients without vertebral fractures and 10 for patients with vertebral fractures) attend GE annually. Gender-specific classes are generally preferred. This is not possible for the nonfracture classes, because only 2-3 men attend these classes each year. Teaching is conducted at three different locations: the outpatient clinic, the athletic facilities at the physiotherapy section, and the training room and kitchen in the occupational therapy section. The purpose of the multifaceted GE is to improve the patient's quality of life by providing information about the disease and counselling on a healthy lifestyle with osteoporosis. The teaching approach is based on lectures and employs methods to ensure active patient involvement. The two programmes contain instruction on disease development, medical treatment, exercise, diet for healthy bones, daily activities, and information about the National Osteoporosis Society. The programmes for patients with vertebral fractures also include pain management, ergonomic demonstrations in the training kitchen, and introduction to an exercise programme for use in the patient's own home.

Patients attending GE are referred either from the outpatient clinic or by the general practitioner and must be diagnosed with osteoporosis to attend GE. Patients unable to participate in physical exercise or suffering from psychiatric diseases or cognitive disturbances are excluded from attending GE.

### 2.2. Participants

Patients from three classes for patients with vertebral fractures (one class for men and two classes for women) and two classes for patients without vertebral fractures (only women) were included, as the selection of classes was intended to represent variation in relation to gender and fracture status. In all, 17 (14 women and three men) of the 26 patients (22 women and four men) accepted to participate in the study. The remaining nine patients who declined participation were four patients with vertebral fractures (three women and one man) and five patients without vertebral fractures; these nine patients had a mean age of 71 years (53 : 85).

The study followed the principles of the Helsinki Declaration [26] and was approved by the Danish Data Protection Agency (ID number 2013-41-2655). According to Danish law, no particular ethical permission was needed to conduct this study. All patients and teachers accepted the audiotaping of GE and the presence of the researcher. The patients and teachers who participated in the study gave written informed consent. All teachers (*n* = 19), eight doctors, three occupational therapists, two nurses, two physiotherapists, two dieticians, and two representatives from the National Osteoporosis Society accepted participation.

### 2.3. Data Collection and Analysis

Participant observation was carried out during GE (approximately 82 hours). This meant that the researcher interacted with the patients and healthcare professionals and constantly tried to be receptive to the experience of the patients studied, the activities, and events [22, 27, 28]. During participant observation, the researcher focused on how imparting knowledge, skills, and recommendations affected the patients' decision-making in relation to (a) how knowledge and preferences were exchanged during GE, (b) whether GE affected their decisions regarding osteoporosis management, and (c) how the patients' individual decision-making was supported during GE. In practice, the researcher was present at the outpatient clinic for about 45 minutes before a session started. She talked to the teachers about the classes of the day, helped them prepare for the session (e.g., turn on computers), and ate lunch with them. On other days, she spent time with patients arriving early and waited in the classroom or outside the athletic facilities of the physiotherapy section. The researcher engaged in informal conversations about the GE programme and daily activities. During classes, the researcher observed the patients, sometimes participating in the conversation or taking part in the class activities. Throughout and after the participant observation, the researcher took field notes on the setting and the interactions taking place. All class sessions were audiotaped (58 hours).

The interviews were carried out just before or during the first week after the start of GE and followed a guide with four themes: (1) patient's everyday life, (2) patient's medical history of osteoporosis, (3) patient's knowledge and understanding of osteoporosis, and (4) patient's expectations of GE. The interviews took place in the patients' homes or in the hospital and were audiotaped.

Data analysis was inductively performed concurrently with data collection and included memo-writing, synthesising, theorizing, and recontextualizing [23, 25]. All interviews and most of the recorded sessions were transcribed verbatim by the first author or a research assistant (955 pages). The analysis focused on the content of the dialogue and interaction between teachers and patients and between the patients and, moreover, on the decisions made or conclusions drawn by the participants. This process of coding led to the generation of preliminary units defining, for example, “individual counselling,” “knowledge and skills to learn,” and “decisions made.” These codes lead to further analysis focusing on the process of decision-making. Data was scrutinized for text units containing words like “decide,” “consider,” “want,” “problem,” “try,” and “attention.” The aim was to check the data and verify the findings, focusing on the characteristics of the patients' considerations and decision-making aspects and how the teachers supported these. Finally, the analysis led to a coherent interpretation of the decision-making process during GE and its impact on the patients' decision-making. The software programme Nvivo10 supported the structuring and analysis of data.

## 3. Results

The patients described their expectations as hoping to “learn something” and “to have the opportunity to get answers to specific questions.” They believed they could make improvements and assumed that attending GE would provide them with clear recommendations for a healthy lifestyle with osteoporosis. They explained that they had never talked thoroughly about osteoporosis with anyone and that they appreciated the opportunity to do so at the GE. Nevertheless, all the patients had reached some kind of understanding about living with osteoporosis depending on how long they had had the disease, the amount of contact they had had with other healthcare professionals, whether they had relatives or friends with the disease, and how much information about osteoporosis they had received. Some patients who had been diagnosed for less than a year when they attended GE expressed they had stopped seeking information because they found the information difficult to understand and intimidating. Instead, they had decided to wait for GE. Patients' demographic and osteoporosis related characteristics are presented in Table 1.

### 3.1. Exchange of Medical Evidence and Personal Experience

During GE, exchange of knowledge consisted of basic medical evidence of osteoporosis provided by the teachers and a high degree of personal experiences from the patients. The educational focus in the dialogue and interaction between and among the patients and teachers was on recommendations to encourage healthy bones and how to implement the recommendations in daily life. We found that recommendations were related to five overall themes: (1) diagnosis and prevention; (2) training and exercise; (3) daily life activities; (4) diet for healthy bones; and (5) medication. The type of recommendation ranged from general principles to specific advice.  Box 1 lists the most frequent recommendations of the teachers in the different sessions in relation to the five overall themes.

The teachers guided the exchange of knowledge, which fostered a dialogue between teachers and patients as well as among patients. Even though providing information on general osteoporosis knowledge and skills was the foundation of GE, the teachers systematically sought and included the patients' individual experiences and needs in the class activities. Multidisciplinary ways of practicing dialogue-based teaching increased the extent of the personal information shared by the patients and encouraged the high degree of knowledge exchange between the teacher and the patients as well as among the patients (Box 2). The patients' personal questions also increased the extent of personal information. For instance, a patient asked the teaching physician the following question:

Can you explain how and why I developed osteoporosis, when I didn't inherit it from my father or mother? F3/d, lesson with physician.

Patients shared experiences related to managing daily life with osteoporosis with each other and with the teachers. For example, in a lesson with the occupational therapist patients described how they managed shopping for staple goods:

When I go shopping, I carry the same amount of goods in each hand. N4/a

I have a rucksack so I can carry the heavy things on my back. N3/a

This brought different personal experiences into play, highlighting both decisional conflicts and predispositions. Therefore, exchange of information was bidirectional and contained basic medical knowledge of osteoporosis provided by the teachers as well as personal experiences from the patients.

### 3.2. Evidence-Based and Experienced-Based Preferences

Patients and teachers expressed their understanding and preferences regarding the recommendations and the implementation of these recommendations in daily life. Patients requested simple and specific answers to questions such as which treatment to choose or whether it was okay to do garden work; however, such answers were seldom offered. Instead, the teachers expressed their opinions and outlined that it was the patients' prerogative. One nurse stated the following:

We are not trying to force you into receiving the treatment [osteoporosis treatment]. We are here to help you.

When teachers expressed their opinions, they highlighted the importance of relying on evidence-based knowledge. It was therefore difficult for the teachers to provide clear and specific answers either because evidence-based information was unavailable or because the recommendations had to be adjusted to match the patients' unique circumstances. For example, a patient considered whether shopping was risky due to pain. She asked the physician if she should give up shopping and the physician answered as follows:

Physician: All I can say is if you want to go shopping and you are able to do so, then I think you should go for it.

F1/c: Okay.

Physician: We can help with painkillers afterwards, but it is entirely up to you whether you find it too hard.

The patients based their preferences on personal experiences, for example, tolerance related to medication and pain. Further, they drew on knowledge from various sources like the Internet, the general practitioner, the pharmacy, family, or friends. Thus, patients only to a certain extent used evidence-based knowledge to confirm their preferences.

Even though the teachers objected to making decisions for the patients, they tried to support them by giving advice and directions on potential avenues that patients could choose to pursue. Likewise, other patients also contributed with advice and tips. Hence, the patients were encouraged to talk to their general practitioner about such topics as obtaining a new DXA scan (a dual energy X-ray absorptiometry, or DXA scan, measures the BMD) or changing the dose of calcium intake. With regard to exercise or daily activities, the patients were told to contact their healthcare centre or were informed about organisations that offered osteoporosis-oriented exercises. A physical therapist offered the following explanation:

You need to contact your local health centre. They offer a lot of different services. I'm sorry, but I can't tell you anymore. These things change all the time.

In a few situations, the teachers offered immediate support to the patients. For example, a dietician asked a patient if she wanted individual counselling. After the session, they went to the consulting room and talked about how the patient could deal with reduced appetite. Another patient decided to change type of osteoporosis medications and asked how to manage this decision. The nurse subsequently arranged an appointment for the patient in the outpatient clinic two weeks later.

The exchange of evidence-based knowledge and personal experiences between teachers and patients led to a mutual deliberation about recommendations and how to lead a lifestyle to ensure bone health. The discussions provided a diversity of answers, opinions, and solutions from which the patients could choose and reaffirmed the patients' responsibility to decide if, when, and how to implement a decision.

### 3.3. Making Decisions Based on a Changed and Personal Understanding

During GE, the patients altered their understanding of an osteoporosis-healthy lifestyle. The GE sessions had an impact on the patients' decision-making in all of the educational themes (Box 3).

Even though GE led to a diversity of answers, it also clarified the opportunities and activities related to specific situations in the patients' lives. One patient stated the following:

They give you tools you can use. There are loads of things I didn't know anything about, but they are all in here now [points at back of head]. I know that habits don't change overnight, it takes much longer, but now I stop and think about my actions more. When I have to lift something heavy now, I think, “Am I doing this right?” I never would have given it a second thought before. F5/d, lesson with nurse.

The increased level of understanding had an impact on patients' decision-making. For example, during GE, the patients learned how to interpret and use the result of a DXA scan. Many of the patients expressed that before attending GE they understood that the DXA scan provided information on disease progression, but they could not read or interpret the results of the scan. Further, they did not know what a* T-score *was (*T*-score describes BMD relative to healthy young bone and is used for diagnosing osteoporosis). Three patients stated the following:

They usually tell me that it is bad, not what it means. N1/a, lesson with physician.

I don't know what a T-score is. F8/e, lesson with the National Patient Society.

I was never told what my T-score showed. U4, interview.

The knowledge exchange about the DXA scans and discussion with the physician helped two patients to decide to postpone starting their medication. They concluded that their risk of fracture was not imminent and believed that the next DXA scan could make them reconsider their decision. A patient without vertebral fractures learned that her BMD had improved. She explained that this knowledge motivated her to make further healthy decisions. Another patient said to the physician that understanding the result of her DXA scan made her realise that improving the *T*-score by taking medicine was important. Further, she explained that other activities that could prevent her from getting a fracture were just as important. She stated the following:

Now I can see the importance of acquiring more healthy habits. N9, lesson with physician.

This patient decided to do more weight-bearing exercises and to be more careful when lifting and carrying objects.

Discussing the recommendations not to lift “too much” or “more than five kilograms” encouraged the patients to describe and discuss their life situations. A patient explained that he considered not carrying his eight-kilogram dog up the stairs any longer. Another patient explained that she had decided only to take the amount of washing powder she needed and not carry the whole five-kilogram container when she was doing her laundry.

A patient who was taking one kind of osteoporosis medicine and previously had been offered a bone anabolic osteoporosis treatment explained the following to the nurse:

The doctor asked whether I could inject myself. I told him “no”, but I really do think he could have told me that the needles were very small and painless. F3/d, lesson with the nurse.

The personal attention allowed the patient to better understand the size of the needles as well as the benefits and costs of the treatment; this had an impact on his decision, as he decided to start the bone anabolic osteoporosis treatment.

All patients voiced the new decisions they had made and even the patients who had had osteoporosis for many years made new decisions. One patient who had been diagnosed with osteoporosis for three years expressed the following:

You can use all the information you get like a dust cloth. You can clean up the habits that aren't healthy, N9/b, lesson with physician.

By exchanging and sharing evidence-based knowledge and personal experiences, the patients changed their understanding of how to manage osteoporosis in daily life. GE did not provide clear recommendations; instead, GE offered solutions and answers about lifestyles conducive to bone health, considering the circumstances of and relevance to the individual patient.

## 4. Discussion 

This study explored multifaceted GE with a particular focus on its impact on patients' decision-making regarding treatment options and lifestyle changes. We have shown that GE engages patients in decision-making as it allows for exchange of knowledge, both basic disease-specific knowledge from the teachers and personal experiences from the patients. Further, GE allows for sharing perspectives among teachers and patients. Studies have shown that the quality of the decision-making process is defined by the extent to which a person recognizes that a decision needs to be made and subsequently engages in the process [12]. Other studies have argued that knowledge about options and possible outcomes along with dialogue about experiences that can be clarified is essential [11, 29]. Thus, GE including the dialogue-based teaching approach may provide a foundation for patients to make better health-related decisions in accordance with their personal values and may thereby reduce decisional conflicts [12, 30, 31].

In our study, patients recently diagnosed with osteoporosis and those who had had the diagnosis for many years participated in the same classes, and both groups improved their understanding of the disease and their attention to treatment and lifestyle, causing them to be more actively engaged in decision-making. This is consistent with previous findings [32] that also demonstrated that attending health education is beneficial to both recently diagnosed patients and patients who have been diagnosed for many years. Based on our study, we suggest that the broad relevance of GE is linked to both new and more experienced patients.

Our study provided information about GE content and about which decisions are considered important and possible to implement in patients' daily life (Box 3). Many alternatives can make the decision-making process more cumbersome, especially if there is limited time for the exchange of medical knowledge and personal experiences [9]. This is particularly problematic when patients meet for a consultation in an outpatient clinic or at the general practitioner [9]. In our study, the amount of time in GE may have been an important prerequisite for the expanded and personal attention patients received and may explain why GE had an impact on patients' decision-making.

We showed that arriving at a decision and determining how to implement it relied on the patients. Even though teachers and patients explored the implications of the decisions and shared their preferences, teachers stressed that it was the patients who ultimately had to make the decision. Thus, teachers therefore refrained from participating in the final step of the decision-making process. There are three issues to consider in relation to this. First, in multifaceted GE, patients often interact with healthcare professionals who are not their primary physician. Even though nearly half of the patients in our study were treated in an outpatient clinic and some knew the teaching nurse or physician, the role of the healthcare professional was different than in a one-on-one consultation. In GE, the role of the healthcare professional is described as that of a teacher or facilitator of knowledge and skills [33]. In the present study the teachers' primary focus was on imparting knowledge and skills related to osteoporosis and not on individual care and medical decision-making. Secondly, decision-making in chronic disease management differs markedly from that of acute disease, which often implies irreversible decisions [9]. In chronic diseases, decision-making concerns not only treatment but also lifestyle. Such decisions may be revisited frequently and it is the patient who has to implement and live with the decisions [9]. Thirdly, during the last 30–40 years, there has been a shift in the patient-provider interaction. The extension of consumerism into healthcare recognizes patients' rights, independence, control, and rationality [2, 34, 35]. This also characterizes the informed decision-making model where the patients make the decision [8]. Together these three issues may explain why the teachers in our study refrained from participating in the last step of the decision-making process and instead emphasized the patients' responsibility to make decisions. Future studies should focus on health care professionals' relationship with patients and what is involved in decision-making and shared decision-making in health care [36].

In our study, patients sought guidance and support concerning decisions as well as implementation. Similar to these findings, a study of patients and professionals in a preventive health program found that patients challenge the effort of healthcare professionals to make patients responsible for their health [37]. In our study, it was mostly the responsibility of the patients to implement decisions. Changing lifestyle and habits is, however, not an easy task. In a few cases, healthcare professionals helped patients to implement a decision, for example, to make an appointment in the outpatient clinic with the purpose of changing medications. This raises the question of whether the supporting role of the teachers should be expanded to include joint actions with the patients and implementing the knowledge and skills acquired in GE. Further, GE may be viewed as a supplement to different kinds of follow-up counselling with general practitioners or other healthcare professional as well as a replacement for individual counselling.

### 4.1. Strengths and Limitations

This study builds on a very extensive data collection compared to most previous studies on multifaceted osteoporosis GE [21, 38–40]. Our study was only in a few cases able to demonstrate that patients implemented the decisions in their daily life. However, according to precaution adoption process model (PAPM), making a decision is an important step towards changing and implementing new behaviour [41]. Conversely, to know whether patients' decisions lead to changes to improve bone health would require further investigation.

This study was based on a specific multifaceted GE program and although the programme described here is similar to other multifaceted GE programs [21], it is not certain that the results can be transferred to GE programmes with different organisation and composition of teachers.

## 5. Conclusion

In this study, we have explored decision-making in the context of multifaceted GE for patients with osteoporosis. During GE, patients changed their understanding of lifestyle conducive to bone health, which had an impact on their decision-making. Patients sought clear recommendations on how to manage a life with osteoporosis and were offered information regarding a variety of ways to follow the recommendations. Teachers supported the patients by providing medical information and listening to patients' experiences. GE led to many healthy decisions on the part of the patients and to advice and directions on how the patients could implement decisions in the future to ensure bone health. Future research is required to investigate if and how patients use their experience from attending GE and how they implement these decisions in daily life.

## Figures and Tables

**Box 1:**
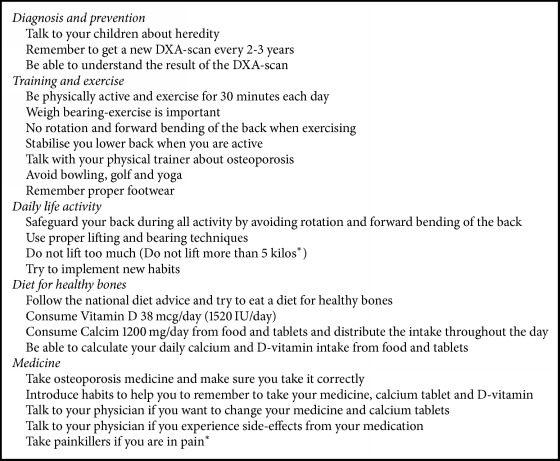
The bone health recommendations originated from GE and related to five overall themes. ^*∗*^Only patients with vertebral fracture; GE = group education.

**Box 2:**
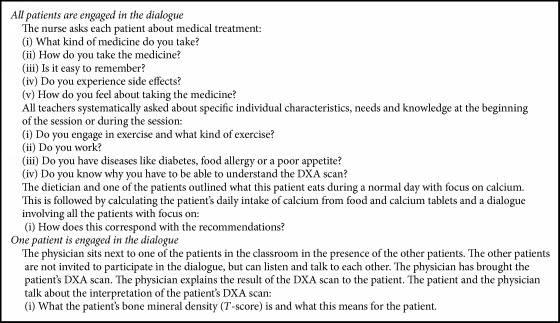
Different ways of practicing dialogue-based teaching during multidisciplinary GE.

**Box 3:**
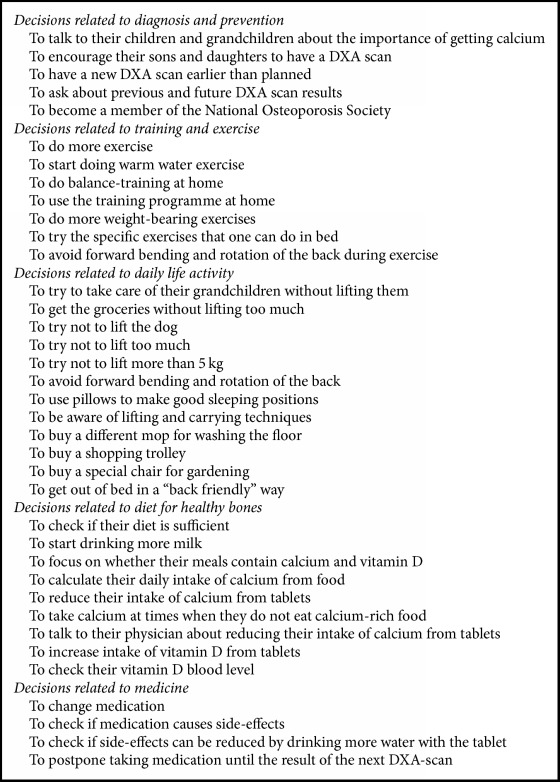
Patients' articulated decisions related to five overall themes.

**Table 1:** Patients' demographic and osteoporosis related characteristics at the end of study period.

Patient ID: F, N/class ID	Age (SD)	Gender	Occupation	Lives alone	Educational background	Years with osteoporosis before GE	Calcium tablets (*n*)	Osteoporosis medication
F1/c	88	Female	Retired	+	Primary school	16	2^a^	Zoledronic acid
F2/c	61	Female	Incapacity benefit	−	Short extensive education	5	0	Denosumab
F3/d	46	Male	Job training	−	Vocational school	1	2	Teripartide^a^
F4/d	71	Male	Retired	−	Primary school	0	2	Teripartide^a^
F5/d	57	Male	Working	+	Vocational school	0	2	Alendronate
F6/e	61	Female	Incapacity benefit	+	Vocational school	0	2	Alendronate
F7/e	62	Female	Incapacity benefit	+	Vocational school	11	2	Zoledronic acid
F8/e	61	Female	Working	−	Bachelor's degree	8	2^a^	Alendronate

F-Mean	63.4 (12.1)							

N1/a	57	Female	Retired	−	Vocational school	0	2	Denosumab^a^
N2/a	53	Female	Working	−	Bachelor's degree	1	2^a^	Alendronate
N3/a	83	Female	Retired	+	Vocational school	5	2	None
N4/a	57	Female	Working	+	Bachelor's degree	5	2^a^	Alendronate
N5/a	69	Female	Retired	+	Bachelor's degree	2	2^a^	None
N6/a	56	Female	Working	−	Bachelor's degree	1	2	Denosumab
N7b	63	Female	Retired	−	Bachelor's degree	0	1^a^	Alendronate
N8/b	56	Female	Working	−	Bachelor's degree	0	1^a^	Alendronate
N9/b	69	Female	Retired	−	Short extensive education	3	1^a	Alendronate

N-Mean	62.5 (9.6)							

F: one or more vertebral fractures; N: no vertebral fracture; SD: standard deviation. ^a^Changed medication, amount of D-vitamin, number of calcium tablets, sort of calcium tablet or time of day for taking calcium tablet type or amount of calcium or in the study period.
